# Testing preference for corn milling coproducts by lactating Jersey cows

**DOI:** 10.3168/jdsc.2025-1008

**Published:** 2026-04-20

**Authors:** K.K. Buse, M.L. Jolly-Breithaupt, K.J. Herrick, A.L. Carroll, P.J. Kononoff

**Affiliations:** 1 Department of Animal Science, University of Nebraska–Lincoln, Lincoln, NE 68583; 2 POET Nutrition, Sioux Falls, SD 57104; 3 Department of Animal Science, Washington State University, Pullman, WA 99163

## Abstract

Manipulations at the intersection of nutrition, behavior, and management may be an important way to apply novel feeding strategies that take advantage of affordable feeds and a cow's natural behavior to increase feed intake. The grain fuel sector continues to modify methods for how corn ethanol is produced. This has resulted in different feed coproducts that can be utilized by the dairy industry. The objective of this study was to determine how preferences of various corn milling coproducts differ and also compare them with corn grain. Eight lactating Jersey cows (169 ± 4 DIM, 29.7 ± 4.55 kg/d milk yield, 21.3 ± 2.78 kg/d DMI; mean ± SD) were utilized. The treatments tested included dried corn distillers grains with solubles (DDGS) in meal form, corn fermented protein (CFP) product in meal form, pelleted corn distillers grains (PEDG), and finely ground corn grain (CRNG). During the experiment, cows consumed a corn-silage-based TMR with 59.8% DM, 17.3% CP, 25.6% starch, and 26.0% NDF. Preference testing was always provided before providing the dairy allotment of TMR. Cows were offered 0.45 kg in each treatment, which was randomly arranged for each cow within their feed bunk. Cows were allowed access to all treatments for 1 h or until the single most preferred feed was fully consumed. Feeds were offered for 9 d with all treatments presented the first 4 d. The most preferred feed was then removed, and the remaining 3 feeds were offered for 3 d. The process was repeated for the last 2 d. Feed preference was ranked from 1 to 4 with 1 being the most preferred and 4 the least. The rankings of each treatment for each cow were summed and averaged. The PEDG was chosen as the most preferred feed (1.00 ± 0.25; mean ± SD) followed by CRNG (2.14 ± 0.38), DDGS (2.50 ± 0.58), and CFP (4.00 ± 0.00). The results were then examined using the Plackett–Luce model to determine the probability a treatment would be chosen first based on the current dataset. The probability of PEDG being selected first was observed to be 98.8% ± 1.60%, whereas the probabilities of CRNG, DDGS, and CFP being selected first were 0.93% ± 0.69%, 0.21% ± 0.73%, and 0.07% ± 0.86%, respectively. Results of this study demonstrate preference is increased over corn when DDGS are pelleted.

## Highlights

• Sequential elimination tests revealed clear cow preference among feeds.
• Lactating Jersey cows preferred pelleted corn distillers and solubles over corn grain.
• Pelleting distillers grains and solubles increased selection compared with meal forms.
• Corn fermented protein was the least preferred among the corn coproduct feeds.

## Graphical Abstract

**Figure:**
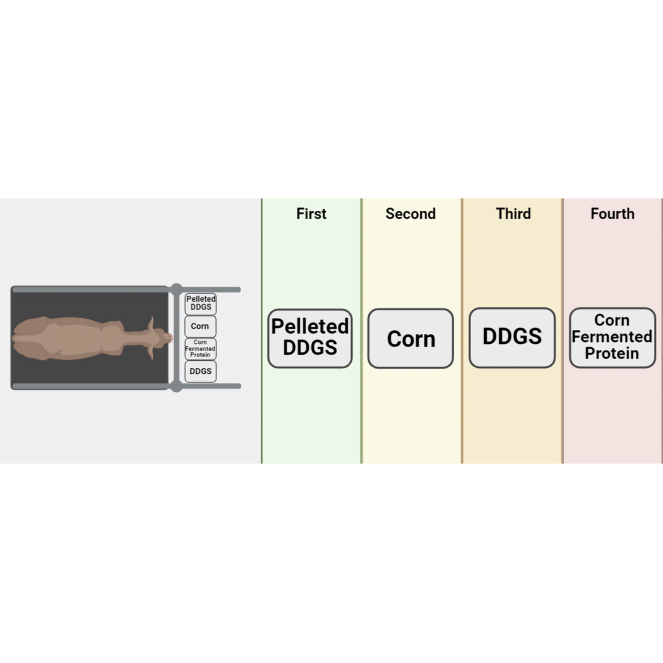
**Summary:**This study explores the preference of individual feedstuffs by dairy cattle. Cows were presented with the choices of ground corn, dried corn distillers grains with solubles (DDGS), a corn fermented protein product of the dry corn milling industry, and pelleted corn distillers grains. Of these options, cows largely preferred the pelleted distillers grains. Although previous research has shown that dried corn distillers grains are highly palatable to cows, altering the form to add bulk density to the feed can allow cows to consume the feed more readily.

For dairy producers, feed is the largest cost of production. Yet, this cost is necessary to maximize milk production as fluid milk and component yield are also the largest portions of income (Wolf, 2003). The increased use of corn milling coproducts often reduces the cost of production because these feeds are often cheaper than corn or other commodities yet they still possess a valuable nutrient profile. Although dried distillers grains with solubles (**DDGS**) are the primary coproduct of ethanol production, the sector continued to evolve with new production methods that bring about differences in chemical and physical composition of the feed. In general, DDGS contain approximately 32% CP and 31% NDF and these nutrients are also highly digestible (Krogstad et al., 2021). A recent modification in the dry milling process involves the separation and concentration of residual protein during the fermentation process resulting in a feed coproduct referred to as corn fermented protein (**CFP**) and contains approximately 52% CP and 36% NDF (Carroll et al., 2023; Shurson, 2023). A pelleted form of DDGS (pelleted corn distillers grains; **PEDG**) may also be produced and aside from physical structure contains similar concentrations of CP (32%) and NDF (31%) (Yoder et al., 2019; Krogstad et al., 2021). This physical modification of DDGS could provide some behavioral benefits by increasing textural interest to the cows, which may encourage feed intake. Pelleting also reduces shrink losses (Knarr et al., 2025) and pelleted feeds are often used as a feed reward in automatic milking systems (Carroll et al., 2024).

The intersection of nutrition, behavior, and management allows for the implication of novel feeding strategies that take advantage of affordable feeds and a cow's natural behavior. It is well known that the most important factor affecting milk yield is DMI (Oba and Allen, 1999). It is advantageous for dairy producers to take measures that encourage cows to maximize intake. The palatability or preference of a feed is a major factor influencing the feed intake of cattle (Baumont, 1996). Observations in previous studies have indicated that PEDG has increasing effects on DMI and although not measured it is possible that animals have a greater preference for PEDG than when DDGS are offered in a meal form (Krogstad et al., 2021). There is a need to better understand the feed preferences of lactating cattle to better understand factors that affect DMI. This is especially the case with automated milking systems that regularly offer pelleted feeds as rewards to incentivize cows to visit milking robots (Carroll et al., 2024). The objective of our study was to determine dairy cattle preference of various corn milling coproducts compared with corn, the parent feed of these coproducts. Because the objective of this experiment was to determine relative feed preference of ingredients, cows were offered fixed quantities of each ingredient and feed disappearance was used solely to determine ranking; total daily DMI of the TMR was not measured as part of this study.

Animal care and experimental procedures were approved by the University of Nebraska–Lincoln Animal Care and Use Committee. Eight lactating Jersey cows (169 ± 4 DIM, 29.7 ± 4.55 kg/d milk yield, 21.3 ± 2.78 kg/d DMI) were utilized in a taste preference experiment. The cows were housed in individual tiestalls equipped with rubber mats in a temperature-controlled (20°C) barn at the Dairy Metabolism Facility in the Animal Science Complex at the University of Nebraska–Lincoln. Cows were milked at 0700 and 1800 h each day. Four days before offering the treatments, cows were trained to eat out of the tubs used in the experiment. The preference test was conducted each day at 1000 h; at this time all feed offered from the previous days was removed and before cows received their daily allotment of TMR. To train animals to become accustomed to eating feed out of the tubs, on d 1 and 2, cows were presented with 4 tubs of approximately 0.90 kg of TMR. Cows were then allowed to eat the TMR from the tubs. Once a cow had finished all 4 tubs, the tubs were removed, and cows were given the remaining daily TMR. Cows were presented with tubs containing 0.45 kg of TMR top dressed with the 4 experimental feed ingredients. Once cows had completely consumed the feed in a single tub, all 4 tubs were removed and the remaining of their daily TMR was provided.

Cows were fed once daily to a target daily refusal rate of 5%. Before feeding cows a TMR, cows were offered tubs containing 0.45 kg of each respective treatment feed from 0930 to 1030 h or until a feed preference was able to be determined for each cow (whichever came first). Tubs were positioned in a horizontal line within the feed bunk of each cow where tubs were placed in a random order. This ensured cows had equal access to each feed. Each feed bunk was separated by a wooden divider, forcing cows to only interact with the tubs placed in front of their specific stall. Both studies lasted 9 d and consisted of 3 feeding segments: feeding segment 1 = d 1 to 4, feeding segment 2 = d 5 to 7, and feeding segment 3 = d 8 and 9. At the end of each feeding segment (d 4, 7, and 9), cow preferences were analyzed and the most preferred feed of that segment was removed. This method, previously published by Erickson et al. (2004), allowed for a sequential ranking of each cow's taste preference from most to least preferred. The individual rankings of each treatment by cow were then averaged to determine the treatment's overall ranking. Using the rankings, the chances of a treatment being chosen first was determined using the Plackett–Luce model in R (v4.0.3; R Foundation for Statistical Computing). A Z-test was also performed using the rankings to determine if the chance an animal would select that given treatment differed from 25% or the probability an animal would select that treatment if there were no preferences. The Plackett–Luce model was used to estimate the relative probability of each treatment being selected first and to evaluate preference differences among treatments. Standard errors of the estimated probabilities were obtained from the model output. Significance was declared with a *P*-value ≤0.05, and trends were observed at *P* ≤ 0.10.

Four different corn products were tested in this experiment and included finely ground corn grain (**CRNG**), DDGS, CFP, and PEDG. POET Bioproducts (Sioux Falls, SD) provided the DDGS (Dakota Gold, POET), CFP, and PEDG (Dakota Gold Pro-Pellet, POET). The PEDG consisted of compressed DDGS pellets approximately 9.5 mm in diameter with a pellet durability index exceeding 90%, produced without binders or additional ingredients, such that the pellets consisted solely of compressed DDGS. Preference for a feed was defined as the first finished tub for each cow or, if no tub was finished, by determining which tub weighed the least (and therefore had the most feed consumed). In cases where cows consumed similar amounts of the feeds and a clear ranking could not be assigned; the observation was recorded as “no preference.” As mentioned previously, for the first 4 d of the study all 4 feeds were offered to each cow. After d 4, the most preferred feed for each cow was removed and 3 remaining feeds were offered for d 5 to 7. After d 7, the second most preferred feed was removed, leaving 2 feeds for d 8 and 9, after which each cow's taste preference was analyzed and ranked from most to least preferred.

The sequential ranking of the offered feeds for each cow is listed in Table 1. Pelleted corn distillers grains was observed to consistently be the most preferred treatment with a mean ranking of 1 (SD = 0). Corn grain was ranked second (2.14, SD = 0.38) with DDGS ranking third (2.5, SD = 0.58) and CFP ranking last (4, SD = 0.52). When cows were provided a choice of all 4 feeds, every cow selected PEDG first. According to the Plackett–Luce, the probability this feed would be chosen first was estimated to 98.8% ± 1.60%, whereas the probability of CRNG, DDGS, and CFP being selected first were 0.93% ± 0.69%, 0.21% ± 0.73%, and 0.07% ± 0.86%, respectively (Table 2). It is noteworthy that in making selection of PEDG, cows completely consumed all feed in the tub and these results serve as strong evidence that PEDG is a highly palatable feed. A potential explanation for this preference is simply that the form of pellets is easier for cows to consume. Cows typically use their tongues to gather and in doing so smaller particles are manipulated into the mouth using the lips while larger particles are drawn into the mouth using the tongue (Beauchemin, 1991). We speculate that cows may prefer PEDG because the physical structure of the feed makes it easier to consume. This same rationale may also explain why CRNG was selected over DDGS and CFP. Although ground corn typically has a smaller particle size than pelleted products, the texture is coarser than that of DDGS and CFP.

**Table 1:** Preferential selection of experimental feeds by cow, ranging from most preferred (1) to least preferred (4)

Item	Treatment^1^			
	CRNG	DDGS	CFP	PEDG
Cow ID				
7047	2	3	4	1
7564	2	—	—	1
7717	3	2	4	1
7837	—	2	—	1
7845	2	—	—	1
8809	2	—	—	1
9173	2	3	4	1
10327	2	—	—	1
Sum	15	10	12	8
Mean	2.14	2.50	4.00	1.00
SD	0.38	0.58	0	0

1 CRNG = finely ground corn grain; DDGS = dried corn distillers grains with solubles; CFP = corn fermented protein; PEDG = pelleted corn distillers grains. No preference indicated by — .

**Table 2:** Estimated probability (%) of each feed being selected first by lactating Jersey cows when all feeds were offered simultaneously, derived from the Plackett–Luce preference model

Item	Treatment^1^			
	CRNG	DDGS	CFP	PEDG
Mean^2^	0.93	0.21	0.07	98.8
SE^3^	0.69	0.73	0.86	1.60
Z^4^	−0.20	−2.21	−3.24	2.83
*P*^5^	0.84	0.03	<0.01	<0.01

1 CRNG = finely ground corn grain; DDGS = dried corn distillers grains with solubles; CFP = corn fermented protein; PEDG = pelleted corn distillers grains.

2 Mean = the estimated percentage chance a diet will be chosen when all treatments are presented.

3 SE = standard error for the percentage chance a treatment will be chosen first.

4 Z = test to determine if the percentage chance of a treatment being chosen first by the animal is different from the percentage chance of no choice at 25%.

5 *P* = indicates that the preference values differ from the percentage chance of no choice at 25%.

Corn DDGS and CFP were the least preferred, with DDGS ranking higher than CFP. Because both feeds have a similar texture, difference in preference was likely due to differences in flavor, scent, or both. Cattle rely on a chemosensation process to recognize chemical signals. In a study evaluating cattle preference for the 4 primary tastes, the bitter taste was recreated using a combination of caffeine and quinine compounds along with urea (Nombekela et al., 1994). One possibility for the decreased preference of CFP could be due to the concentration of protein in the product. Another possibility is that undesirable flavors or odors were imparted on the product during production that would cause cows to select against it, which has been seen in a study comparing high protein DDGS and soybean meal (Brown and Bradford, 2020). Because the CFP did not contain distillers solubles, it is also possible that the lack of this constituent of the dry milling process made it less appealing to the cows. The findings of this study further advance our understanding of feed behavior in dairy cattle and indicate that physical form influences feed selection behavior, with cows consistently selecting pelleted distillers grains and solubles over the other feeds offered. Future research should investigate the potential of a chemical basis for the observed aversion for CFP. Although this experiment evaluated palatability rather than intake or production, previous work suggests responses to pelleting may depend on diet characteristics such as forage concentration and particle size. In those studies, pelleting DDGS did not affect milk yield, energy-corrected milk, or milk fat production, although an interaction with forage concentration was observed for milk protein yield, which increased when pelleted DDGS were fed in higher-forage diets. The preference observed here reflects improved palatability and physical accessibility of pelleted feeds. From a practical perspective, pelleting also improves feed handling and bulk density; industry estimates suggest pelleting adds approximately $4 to $6 per tonne.

## References

[bib1] Baumont R. (1996). Palatability and feeding behaviour in ruminants. A review. Ann. Zootech..

[bib2] Beauchemin K.A. (1991). Ingestion and mastication of feed by dairy cattle. Vet. Clin. North Am. Food Anim. Pract..

[bib3] Brown W.E., Bradford B.J. (2020). Effects of a high-protein corn product compared with soy and canola protein sources on nutrient digestibility and production responses in mid-lactation dairy cows. J. Dairy Sci..

[bib4] Carroll A.L., Fincham G.M., Buse K.K., Kononoff P.J. (2024). Feed preference in lactating dairy cows for different pellet formulations. JDS Commun..

[bib5] Carroll A.L., Morris D.L., Jolly-Beithaupt M.L., Herrick K.J., Watson A.K., Kononoff P.J. (2023). Energy and nitrogen utilization of lactating dairy cattle fed increasing inclusion of a high-protein processed corn coproduct. J. Dairy Sci..

[bib6] Erickson P.S., Davis M.L., Murdock C.S., Pastir K.E., Murphy M.R., Schwab C.G., Marden J.I. (2004). Ionophore taste preferences of dairy heifers. J. Anim. Sci..

[bib7] Knarr L.E., Bowen K.M., Lynch E.A., Estanich E.B., Renner A.K., Adejumo R.O., Ferrel J., Moritz J.S. (2025). Throughput agents alleviate concerns about pelleting broiler feed with low-moisture corn and large pellet die thicknesses. J. Appl. Poult. Res..

[bib8] Krogstad K.C., Herrick K.J., Morris D.L., Hanford K.J., Kononoff P.J. (2021). The effects of pelleted dried distillers grains and solubles fed with different forage concentrations on rumen fermentation, feeding behavior, and milk production of lactating dairy cows. J. Dairy Sci..

[bib9] Nombekela S.W., Murphy M.R., Gonyou H.W., Marden J.I. (1994). Dietary preferences in early lactation cows as affected by primary tastes and some common feed flavors. J. Dairy Sci..

[bib10] Oba M., Allen M.S. (1999). Evaluation of the importance of the digestibility of neutral detergent fiber from forage: Effects on dry matter intake and milk yield of dairy cows. J. Dairy Sci..

[bib11] Shurson, G. C. 2023. USGBC High Protein DDGS Handbook. The U.S. Grains & BioProducts Council.

[bib12] Wolf C.A. (2003). The economics of dairy production. Vet. Clin. North Am. Food Anim. Pract..

[bib13] Yoder A.D., Jones C.K., Herick K.J., Paulk C.B., Bradford B.J., Stark C.R. (2019). Effects of low oil DDGS on pellet quality and pellet mill motor electrical efficiency. Appl. Eng. Agric..

